# More than just lipid balls: quantitative analysis of plastoglobule attributes and their stress-related responses

**DOI:** 10.1007/s00425-022-03848-9

**Published:** 2022-02-10

**Authors:** Miren I. Arzac, Beatriz Fernández-Marín, José I. García-Plazaola

**Affiliations:** 1 Department Plant Biology and Ecology, University of the Basque Country (UPV/EHU), Barrio Sarriena s/n, 48940 Leioa, Spain; 2 Department Botany, Ecology and Plant Physiology, University of La Laguna (ULL), 38200 Tenerife, Spain

**Keywords:** Chloroplast, Plastoglobule, Stress response, Transmission electron microscopy, Thylakoid, Ultrastructure

## Abstract

**Main conclusion:**

**Plastoglobules are ubiquitous under non-stress conditions and their morphology, closely related to their composition, changes differently depending on the specific stress that the plant undergoes.**

**Abstract:**

Plastoglobules are lipoprotein structures attached to thylakoid membranes, which participate in chloroplast metabolism and stress responses. Their structure contains a coating lipid monolayer and a hydrophobic core that differ in composition. Their function in chloroplasts has been studied focussing on their composition. However, we currently lack a comprehensive study that quantitatively evaluates the occurrence and morphology of plastoglobules. Following a literature search strategy, we quantified the main morphological attributes of plastoglobules from photosynthetic chloroplasts of more than 1000 TEM images published over the last 53 years, covering more than 100 taxa and 15 stress types. The analysis shows that plastoglobules under non-stress conditions are spherical, with an average diameter of 100–200 nm and cover less than 3% of the chloroplast cross-section area. This percentage rises under almost every type of stress, particularly in senescence. Interestingly, an apparent trade-off between increasing either the number or the diameter of plastoglobules governs this response. Our results show that plastoglobules are ubiquitous in chloroplasts of higher plants under non-stress conditions. Besides, provided the specific molecular composition of the core and coat of plastoglobules, we conclude that specific stress-related variation in plastoglobules attributes may allow inferring precise responses of the chloroplast metabolism.

**Supplementary Information:**

The online version contains supplementary material available at 10.1007/s00425-022-03848-9.

## Introduction

Since the first description of chloroplasts by Hugo von Mohl in 1837, the characterisation of the complex structure of these organelles has run in parallel with the advancements in microscopy (Staehelin 2003). In this endeavour, the definitive milestone was the development of the electron microscopy in the 50s, a technique that allowed enough spatial accuracy to observe chloroplast ultrastructure at 1 Å resolution (for reviews on chloroplast ultrastructure, see: Staehelin 2003; Kirchhoff 2019). One of the structural features typically observed in these organelles are the plastoglobules (PGs). PGs are spherical lipoprotein particles found in plastids, which are attached to thylakoid membrane zones of high curvature in chloroplasts (Austin II et al. 2006). Under TEM, PGs usually appear darker than the surrounding stroma because of their highly osmiophilic character. Until recently, they were considered as simple passive storage organelles (Lichtenthaler and Peveling 1967; Steinmüller and Tevini 1985; Kessler et al. 1999) and its presence was barely reported or quantified in ultrastructural studies. However, the characterisation of its proteome in 2006 revealed an unexpectedly active metabolic role (Vidi et al. 2006; Ytterberg et al. 2006). Since then, the physiological roles of PGs have been the subject of several reviews (Bréhélin and Kessler 2008; Eugeni Piller et al. 2012; Nacir and Bréhélin 2013; Rottet et al. 2015; van Wijk and Kessler 2017; Wójtowicz and Gieczewska 2020; Michel et al. 2021).

The existence of PGs is a well-preserved trait across the evolution of photosynthetic eukaryotes (Lohscheider and Río Bártulos 2016). Thus, PGs have been spotted in all the branches of the evolutionary tree of oxygenic photosynthetic organisms, including, among others, chlorophytes (Holzinger et al. 2011a; Procházková et al. 2021), phaeophytes (Holzinger et al. 2011b), rhodophytes (Schmidt et al. 2012), cryptophytes (Laza-Martínez et al. 2012), diatoms (Balamurugan et al. 2017) and all groups of land plants. Even cyanobacteria present a type of lipid droplets in the cytoplasm that are comparable to eukaryotic PGs (van de Meene et al. 2006). However, for the sake of simplicity, in the present study the term PGs will be used only to refer to those lipid bodies located in photosynthetically active eukaryotic chloroplasts, thereby excluding chromoplasts and cytosolic lipid droplets.

**Fig. 1:**
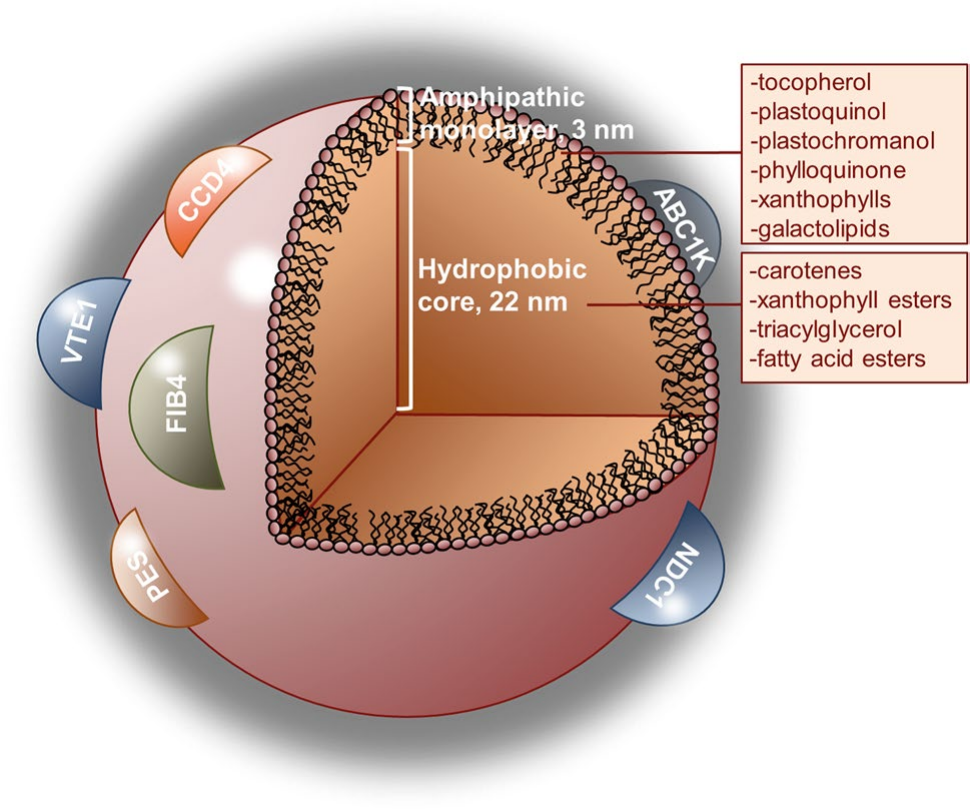
Structure and composition of a model plastoglobule. The external and amphipathic coating monolayer of lipids contains several proteins with relevant roles in chloroplast metabolism, as well as different components of thylakoid membranes. The hydrophobic internal core contains mostly neutral lipids, including some storage molecules such as triacylglycerol. ABC1K, activity of BC1 complex kinase; CCD4, carotenoid cleavage dioxygenase 4; FIB4, fibrillin 4; NDC1, NADP(H) dehydrogenase C1; PES, phytol ester synthase; VTE1, tocopherol cyclase

Plastoglobules are comprised of a surrounding lipid monolayer coat and a hydrophobic core (Fig. 1). The PG metabolome includes different types of lipids (Tevini and Steinmüller 1985) that can be grouped in two main categories: (i) neutral lipids, which are stored in the hydrophobic core of the PG (Lundquist et al. 2013; Rodríguez-Concepción et al. 2018) and (ii) amphipathic lipids, located in the surrounding lipid coat (Zbierzak et al. 2010; Rodríguez-Concepción et al. 2018). Some of these metabolites result from the degradation or remodelling of thylakoids and photosynthetic apparatus, while others are actively synthesised in the PGs (van Wijk and Kessler 2017). Among them, prenyl lipids [including plastoquinone-9 (PQ-9), tocopherol, plastochromanol-8 (PC-8) and phylloquinone] represent a large fraction in PGs (Steinmüller and Tevini 1985). These molecules have protective roles under stress conditions (Hussain et al. 2013; Havaux 2020), and some of them participate in the electron transport chain, as is the case of phylloquinone (Biggins et al. 1990) and plastoquinone-9 (Van Eerden et al. 2017). This pool of prenyl quinones could help maintaining the redox balance of the chloroplast through their mobilisation and exchange with the ones in the thylakoids as a rapid way of preventing photo-oxidative damage, as it has been already proposed for PQ-9 (Szymańska and Kruk 2010; Zbierzak et al. 2010).

The PG proteome is composed of approximately 30 core proteins and some others that are recruited under specific conditions (van Wijk and Kessler 2017). They are localised in the periphery of PGs, probably attached to the monolayer by hydrophobic domains (Ytterberg et al. 2006). Among them, some major functional groups can be identified (Lundquist et al. 2012; Nacir and Bréhélin 2013): structural proteins, kinases, transporters and enzymes involved in the breakdown of carotenoids, chlorophyll and lipids, and in the biosynthesis of jasmonate, carotenoids and prenylquinones. Overall, PG metabolome and proteome can be integrated in four major physiological modules (Lundquist et al. 2012; Michel et al. 2021): chlorophyll degradation, thylakoid remodelling, biosynthesis of prenylquinones and carotenoid metabolism. Within their proteome some of the identified proteins are: tocopherol cyclase (VTE1), involved in the biosynthesis of tocopherol and plastochromanol-8 (Porfirova et al. 2002; Vidi et al. 2006); NAD(P)H dehydrogenase C1 (NDC1) that reduces the plastoquinone pool in PGs (Eugeni Piller et al. 2011); carotenoid cleavage dioxygenase (CCD4) implicated in carotenoid catabolism (Huang et al. 2009); and phytol ester synthase 1 (PES1) and 2 (PES2), which participate in the formation of phytyl ester derived from chlorophyll and of fatty acids derived from galactolipids (Lippold et al. 2012). In addition, it must be included the fibrillin family proteins (FBN), some of the most abundant proteins found in PGs that are considered to have a variety of biological functions, for instance structural proteins, associated with transport of metabolites or involved in response to stress (Singh and McNellis 2011). This turns PGs into keystones of chloroplast metabolic architecture, participating in several fundamental processes related to plant development and to environmental responses.

Several studies have described that the quantity and size of PGs increase in response to many types of environmental stresses (reviewed in Venzhik et al. 2019). This is for example the case of temperature changes (Zhang et al. 2010), leaf senescence (Hörtensteiner 2006), salinity (Naidoo et al. 2011), metal toxicity (El-Banna et al. 2019), pathogens (Raman et al. 2006), high light (Lichtenthaler et al. 1981) or desiccation (Pressel and Duckett 2010; Fernández-Marín et al. 2013). Such active involvement of PGs on the metabolic response to stress relies on their diverse functional and metabolic roles, and so, several systemic responses to stress are somehow connected with the PG metabolic network. For example, the readjustment of light harvesting capacity, a widespread response to almost all types of environmental stress, is also connected to the PGs by their involvement in the biosynthesis of carotenoids (Rodríguez-Concepción et al. 2018), as well as in their catabolism or storage, particularly during senescence and severe stress periods (Lippold et al. 2012; Espinoza-Corral et al. 2021).

As mentioned in the above paragraphs, a causal relationship between environmental stress and PGs can be discerned by observational and functional approaches. Besides, PGs have also been found in chloroplasts from non-stressed plants where they perform essential metabolic activities (Bréhélin and Kessler 2008; Lundquist et al. 2013; Rottet et al. 2015). Despite these scattered evidences found in the literature, we currently lack a comprehensive study that quantitatively evaluates the occurrence of PGs and their attributes in chloroplasts of non-stressed plants, as well as the magnitude and trend of changes of these attributes under stress conditions. In this context, through the present literature survey, we aim to provide, from a morpho-functional perspective, a quantitative response to two basic questions: (1) What the PG average number and ultrastructural attributes in non-stressed chloroplasts are, and (2) whether and how (size and direction) these ultrastructural properties may change in response to different stress types. For that purpose, after a literature survey, we have quantitatively re-analysed more than 1000 TEM images published during the period 1967–2019, covering a wide range of environmental stresses and phylogenetic groups.

## Materials and methods

### Data search and selection criteria

We conducted a systematic publication search in the database Web of Science for studies measuring the effects of physical or biological stresses on the number and size of plastoglobules until the year 2019. The search terms used were: “plastoglob* AND (leaves OR leaf) AND article [Document Type] AND science/technology [Research Domains]”. 445 publications that met these criteria were individually pre-evaluated as potential candidates for review. Based on the information included in the abstract, only research articles in which the measurements were conducted on chloroplasts of photosynthetic tissues were included, excluding those focussing on other types of plastids, such as fruit and floral tissues. With these restrictions, 291 articles were finally used for data analysis. The search criteria employed in this study has probably overlooked many articles where PGs were analysed but not considered in the title or the abstract, as well as articles where the data were not presented as TEM images.

### Data extraction and processing

In the articles evaluated for the present study, two sources of data were employed: (i) measured values were obtained after the analysis of TEM images, and (ii) reported values were collected on tables or graphs. The first source was used for the quantitative analyses while the second was used exclusively for data validation. For some articles, more than one dataset was obtained, depending on the number of species or stress type/intensity levels studied. The following criteria were used for excluding TEM images from processing and analyses: (i) less than 80% of the chloroplast surface was visible, and (ii) the image contrast and resolution was not enough to delimitate PGs from the background. In turn, whenever a single image showed several chloroplasts, a maximum of four representative of the sample were selected for the analysis. This prevented an overrepresentation of single biological replicates. All images from the articles were analysed with ImageJ 1.5 software (Wayne Rasband, National Institutes of Health, Bethesda, MD, USA). To calculate sizes/areas, we measured the number of pixels and transformed them into μm, based on the original scales provided by the authors in the figures. Moreover, the mean grey value obtained by the software was used for electron density estimation.

From the TEM images present in the selected articles (examples of different PGs found during the study are shown in Supplementary Fig. S1), we measured the following parameters: chloroplast cross-section area (CA), PGs total area, number of PGs per chloroplast cross-section (NPG), the area occupied by starch granules, level of aggrupation of the PGs, PGs mean grey value and vacuole mean grey value (as background reference). The analysis of the level of aggrupation was estimated counting the number groups of PGs per chloroplast, considering a group those where more than two PGs were close enough to be connected with each other. The raw data were further processed to obtain the area and diameter of individual PGs (ØPG), the percentage of chloroplast area (calculated as: Chloroplast cross-section area − Starch granules area) occupied by PGs total area (%PG) and the normalised electrodensity (nED), calculated as: (reference mean grey value − PGs grey mean value)/reference mean grey value × 100. Furthermore, assuming the thickness of the lipid coat to be 3 nm, the relative areas of the coat and the core were calculated. From these areas, we obtained the percentage of the chloroplast area (obtained as in %PG) occupied by the total area of each part (from now on referred to as %Core for the core and %Coat for the coat), as well as the ratio between them, obtained as: %Core/%Coat. For those photographs in which the scale was not reported, only the %PG and the nED were calculated. Finally, the obtained data were re-checked to remove any unusual or erroneous values generated by image analysis. For this, a box-plot diagram was generated and all the outliers in each category were individually reconsidered. A datum was considered as an outlier when its value was more than 1.5 times the interquartile range (IQR) above the third quartile or below the first quartile (Tukey’s method).

Aside from image analyses, from each publication, the following information was collected: analysed species, type of tissue, applied stress and growth conditions (temperature, photosynthetic photon flux density (PPFD) and photoperiod). All the data obtained in the study can be found in the supplementary material.

**Fig. 2:**
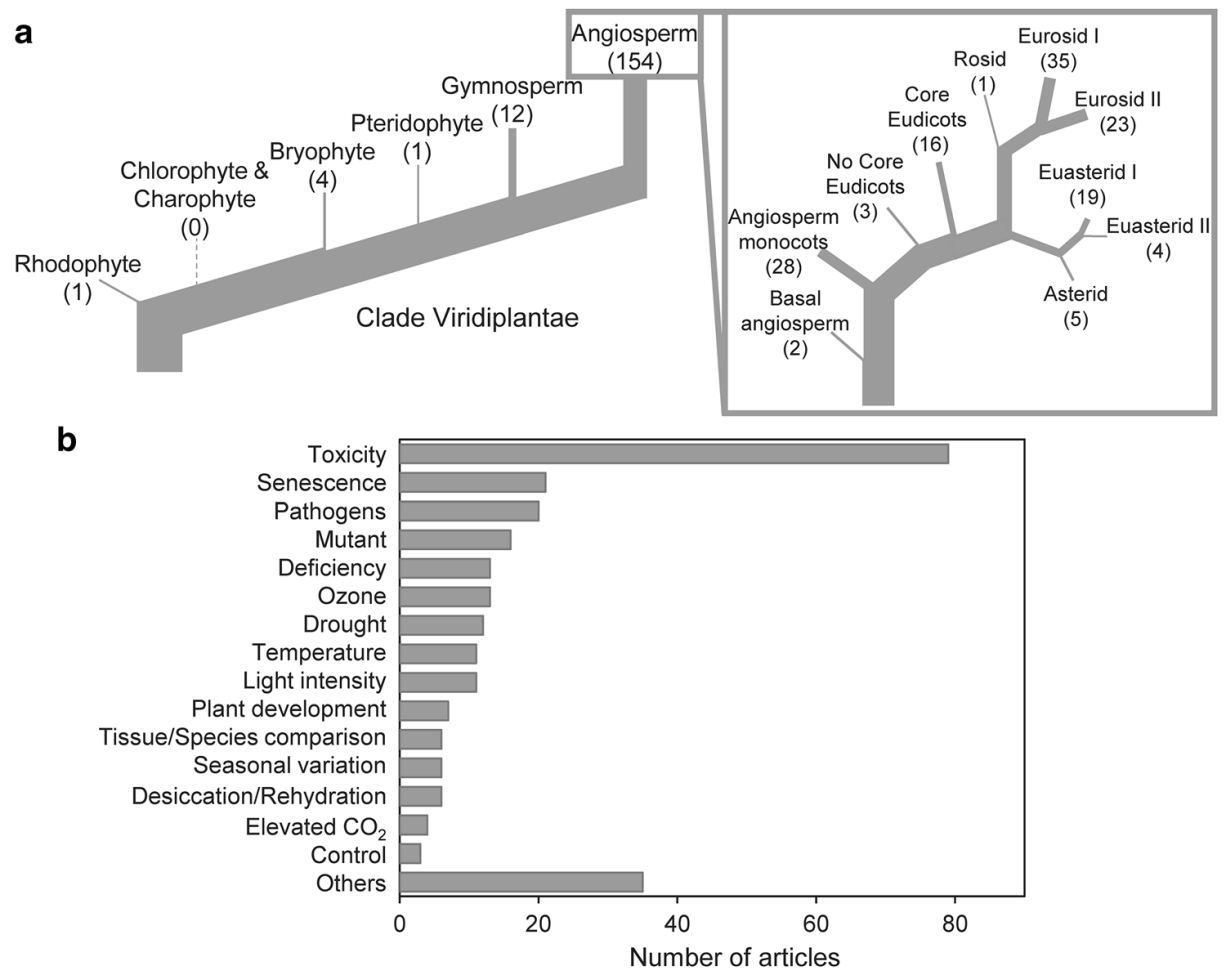
Summary of taxons and stresses covered in this quantitative review. **a** Distribution of the analysed articles according to the taxonomic group. **b** Distribution of the analysed articles according to the stress type, as described by the authors. In panel **a**, the values in parentheses indicate the number of species of the quantitative review that corresponds to each group

The extracted data were categorised as “non-stress” or “stress” conditions following the criteria of the original authors. Within each article, different stress treatments were considered individually and hereafter referred to as cases. In the articles where a non-stress was not indicated by the authors, all the conditions were considered as stress. Based on the details provided in the article, we classified all stresses in 16 types (all of them are enumerated in Fig. 2 and the most common explained in Supplementary Table S1). In the present study, we have grouped all the treatments considered as “stress factors”. Among them, senescence was also considered a stress even though sensu stricto it is not an experimental condition because it strongly affects plant function. Further processing of the data consisted of two separate analyses: (i) a characterisation of PGs attributes under non-stress conditions, for which, we analysed non-stress data only, and (ii) an assessment of the changes in PGs attributes undergone under stress conditions, for which, the effect size (ES) was measured as follows:

1 $$ES=\mathrm{ln}\left(\frac{{\overline{X} }_{Stress}}{{\overline{X} }_{Control}}\right),$$

where $\overline{X}$ is the mean of the values obtained from all the chloroplast analysed for a determined stress (${\overline{X} }_{Stress})$ or non-stress condition (${\overline{X} }_{Control}$). When only stress conditions were reported in an article, the lower stress intensity value was identified and considered as the non-stress for applying this equation.

### Statistical analyses

The difference in the response between non-stress and stress conditions was analysed using Kruskal–Wallis test. Normality was checked with Shapiro tests. Homogeneity of variances was checked with Levene’s test. When the homogeneity of variances was not fulfilled, data were analysed using generalised least squares (*gls*) and general linear hypothesis (*glht*) as a post hoc. The relationship between data presented in tables and data obtained from figures was analysed using a linear regression. The correlation of the data was measured by Kendall correlation coefficient, and the slopes of the lineal regression and the hypothetical 1:1 relation were compared using the least-square method. All the analyses were performed with the statistical programming environment R 4.0.3 (R Core Team 2018), using the *car* (v3.0–10; Fox and Weisberg 2019), the *multcomp* (Hothorn et al. 2008) and the *lsmeans* (v2.30–0; Lenth 2016) packages. Principal component analysis (PCA) was performed using the *factoextra* package (v1.0.7; Kassambara and Mundt 2020).

## Results

### Overall picture of the data and method validation

Present literature survey has analysed a total of 1094 TEM images from 255 articles published between 1967 and 2019. These images correspond to 153 species, representing angiosperms the majority of observations (88.31%), followed by gymnosperms (7.79%) (Fig. 2a). The taxonomic diversity among angiosperms was reasonably well represented. Among them the groups more frequently depicted were monocots, core eudicots, eurosids I and II and euasterids I, with the highest number of records corresponding to model and crop species, such as *Arabidopsis* *thaliana*, *Nicotiana* *tabacum*, *Hordeum* *vulgare* or *Oryza* *sativa.* In the case of gymnosperms, the recurrence of species in the articles was higher (among 26 studies, only 12 species were represented). Based on our criteria of classification, more than 20 stress types were identified (Fig. 2b), being the stresses categorised as “toxicity” treatments the most frequently reported (30%). Notably, more than half of the considered stress types were present in less than ten articles (Fig. 2b).

The validity of our calculations was tested in those articles that reported simultaneously quantitative values (i.e. Tables) and TEM images (Supplementary Fig. S2). For this purpose, we only considered the cases where both values were present in the article for the same species and treatment level. The statistical analysis performed showed that the linear correlation between reported and calculated values was for all parameters positive and significant, with slopes of the regression line ranging between 0.69 and 1.30 (average 0.94 ± 0.11, *n* = 5). However, the slope of the regression differed significantly from the expected 1:1 relation slope (*P* < 0.05) for the chloroplast area, diameter of PG and percentage of PG. Overall, the linear model was not a 1:1 relation for any of the parameters analysed, but on average was very close to it, thereby validating our approach of data analysis.

### Attributes of PGs under non-stress conditions

**Fig. 3:**
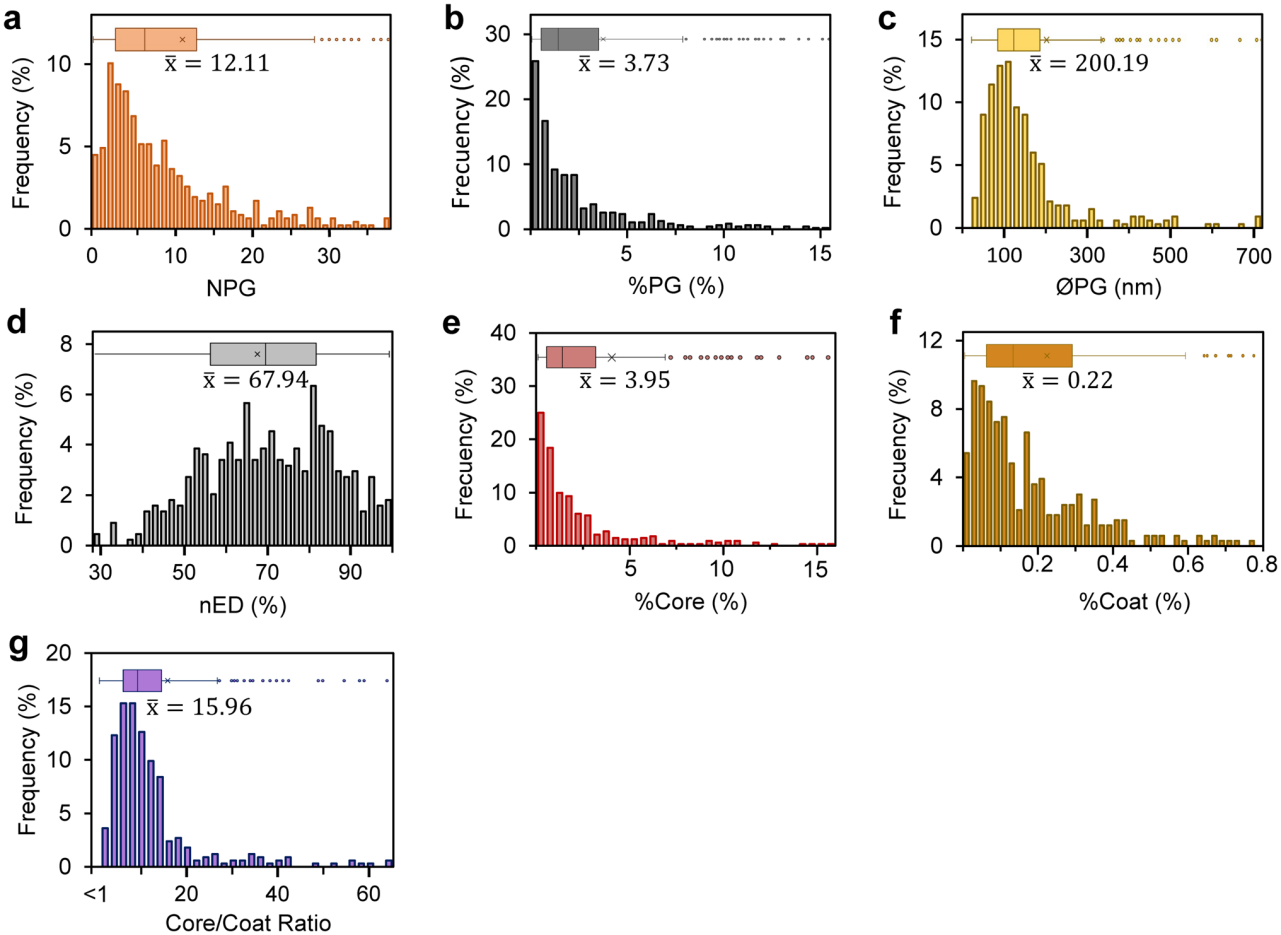
Frequency distribution of the main attributes of PGs under non-stress conditions. **a** Number of PGs per chloroplast cross-section (NPG). **b** Percentage of the chloroplast cross-section area occupied by PG (%PG). **c** Diameter of PG (ØPG). **d** Normalised electrodensity of PG (nED). Higher value on the scale indicates more osmiophilicity of the PG. **e** Percentage of the chloroplast area occupied by total core area (%Core). **f** Percentage of the chloroplast area occupied by total coat area (%Coat). **g** Ratio between the %Core and %Coat. The values indicated on the X-axis correspond to the interval where 95% of the total data are located. Boxplots display the distribution of the whole dataset per attribute, the central line represents the median, the cross represents the mean and the whiskers represent the minimum and maximum values of non-atypical data

The frequency histograms of the most relevant parameters for the characterisation of non-stress PGs are shown in Fig. 3. All the calculated parameters were non-normally distributed with a skew towards higher values than the median, except for colour intensity (Fig. 3d). All the parameters, excluding nED, had around 7–12% of outliers (based on the Tukey method). These outliers caused the mean to diverge to higher values than the median. For example, the average NPG was around 12 and the median was around 7 (Fig. 3a). In this case, some outliers presented more than 100 PGs per chloroplast section, diverting the mean almost to double the value of the median.

Under standard non-stress conditions, 75% of the analysed chloroplasts contained between 1 and 14 PGs (Fig. 3a), being 2 PGs the most frequent value. Virtually, all chloroplasts contained PGs; being absent in just in a few cases (4.5%) (Fig. 3a). In 75% of the analysed images, the %PG was lower than 3.4% of chloroplast area, except for the outliers in which this parameter can be higher than 10% (Fig. 3b). The mean of ØPG was around 200 nm for most cases (Fig. 3c), whereas the median was 121 nm. Although less frequently, some larger PGs, with more than 1000 nm diameter (2%) were found (mainly in *Vitis* *vinifera*, *Fagus* *sylvatica*, *Quercus* *suber*, *Chromolaena* *odorata* and *Kosteletzkya* *virginica*), while the minimum ØPG was 20 nm (mainly in *Nicotiana* *tabacum*, *Citrus* *sinensis*, *Hedyotis* *verticillata* and *Leptochloa* *chinensis*) (Fig. 3c). Regarding the nED, all PGs were more osmiophilic than the vacuole used as reference (Fig. 3d). Indeed, 85% of the cases were above the 50% value of our scale. Finally, the %Core was always higher than the %Coat (Fig. 3e–g). In half of the cases studied, the core/coat ratio was around 6–14-fold, being the median 9.4-fold. What is more, no cases were reported where the ratio was smaller than one, and only in 5% of the values the ratio was smaller than 3.5-fold.

**Fig. 4:**
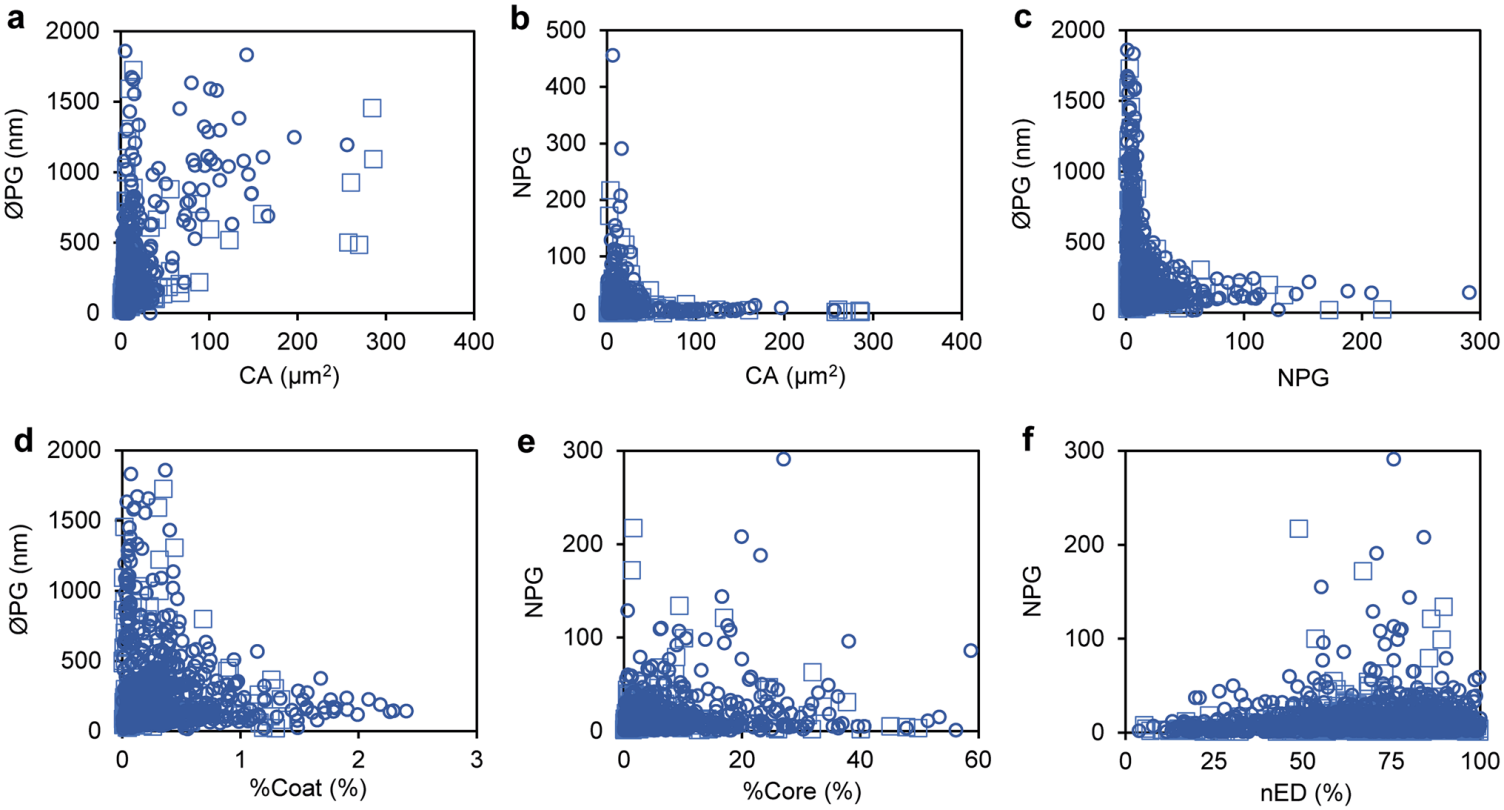
Main relationships between the parameters analysed in the study. **a** Diameter of PG (ØPG) compared to chloroplast cross-section area (CA). **b** Number of plastoglobule per chloroplast cross-section (NPG) compared to CA. **c** ØPG compared to NPG. **d** ØPG compared to the percentage CA occupied by total coat area (%Coat). **e** NPG compared to the percentage CA occupied by total core area (%Core). **f** NPG compared to normalised electrodensity (nED). The circles represent the values of stress conditions and the squares the values of non-stress conditions

After this, we analysed the relationships between all the evaluated parameters. Based on our criteria, the most relevant relationships are presented in Fig. 4 and the rest of relations are shown in Supplementary Fig. S3. PGs of non-stress and stress conditions showed the same tendencies in all parameters. Large chloroplasts (higher than 10 µm of diameter) did not show neither a higher number of PGs nor the presence of larger PGs (Fig. 4a, b). Conversely, the presence of high number of PGs or of large PGs was only found on small chloroplasts (smaller than 2 µm of diameter). Likewise, the relationship between ØPG and NPG showed that large PGs were only present in a small amount, whereas when present in high number, PGs were always small sized (Fig. 4c). Accordingly, the relative proportion of core and coat followed also this characteristic tendency. The %Coat was proportionally higher only when PGs were small-sized (Fig. 4d), whereas the %Core increased in the cases where NPG was lower (Fig. 4e). Finally, the few cases where nED was low corresponded to small values of NPG (Fig. 4f).

### Responses to stress

**Fig. 5:**
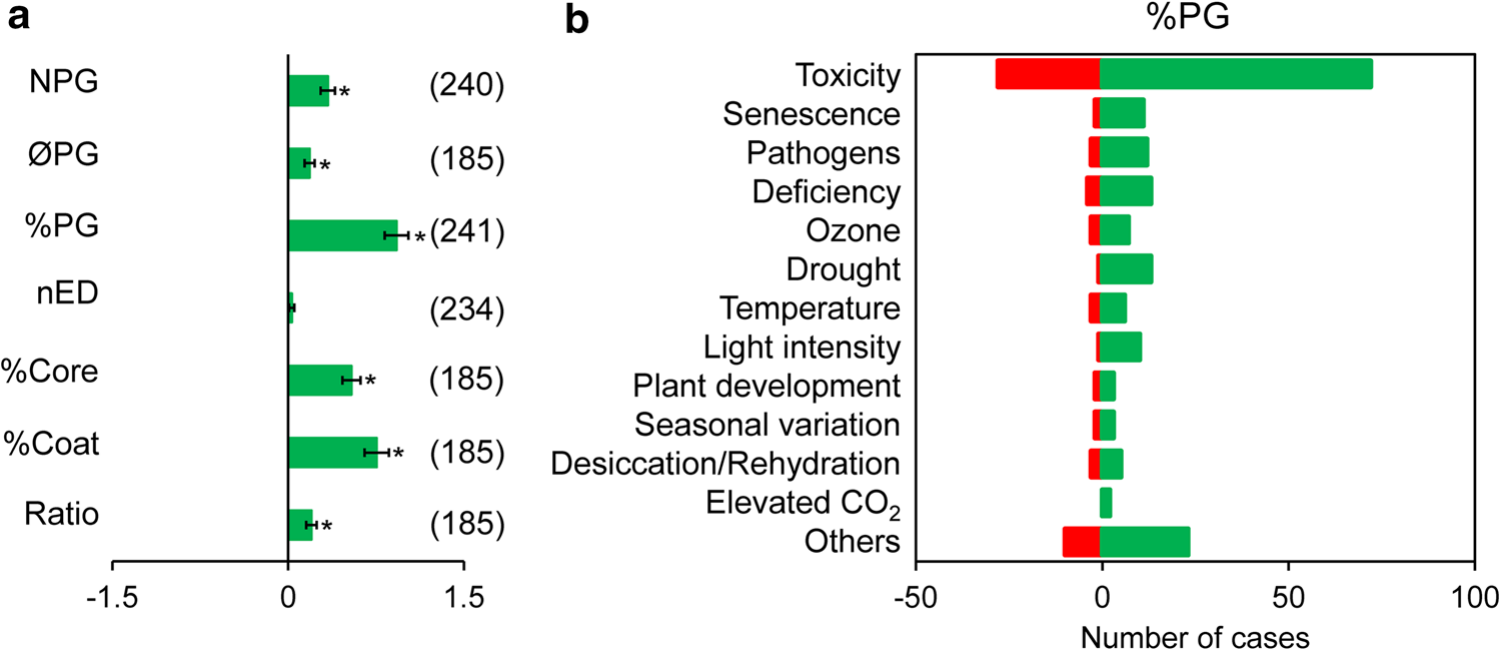
PGs response to stress conditions. **a** Effect size of stress on the seven parameters of the PGs selected. All stress types studied were considered together. Data were calculated as: ln(X_*Stress*_/X_*Control*_). Each bar and whiskers represent the mean ± SE (*n* indicated within brackets for each parameter). **b** Effect of selected stresses on the %PG. Green colour indicates increment of the parameter values under stress, and red colour indicates decrease of the parameter values under stress. Each value was considered non-stress or stress based on the criteria of each article’s authors. Notice that the difference on the number of cases among parameters is due to the availability of a size scale in the pictures and to the quality of the images. Statistically significant differences are indicated by the asterisks (*P* < 0.05). nED, normalised electrodensity; NPG, number of PGs per chloroplast cross-section; Ratio, %Core/%Coat ratio; ØPG, diameter of individual PG; %Coat, percentage of chloroplast cross-section area occupied by the total coat area; %Core, percentage of chloroplast cross-section area occupied by the total core area; %PG, percentage of chloroplast cross-section area occupied by the total PG area

The general patterns of response to stress were analysed employing the effect size (ES) of each factor. When all the stress factors were considered together, a general tendency of increase was observed for all the PG attributes (Fig. 5a), being particularly remarkable in the case of %PG and the NPG. Interestingly, the increase of %Coat was higher than that of %Core. The only parameter that was not significantly affected by stress was nED. We selected %PGs, the parameter with the strongest responsiveness, to visualise the distribution of the values that result in this increase (Fig. 5b). Overall, %PG increased in most cases irrespective of the stress type (Fig. 5b). However, it should be noted that there were examples of %PG decrease for all factors considered. For example, in toxicity, 28% of the cases experienced to some extent a decrease on the %PG, or in deficiency, these cases were the 23%.

**Fig. 6:**
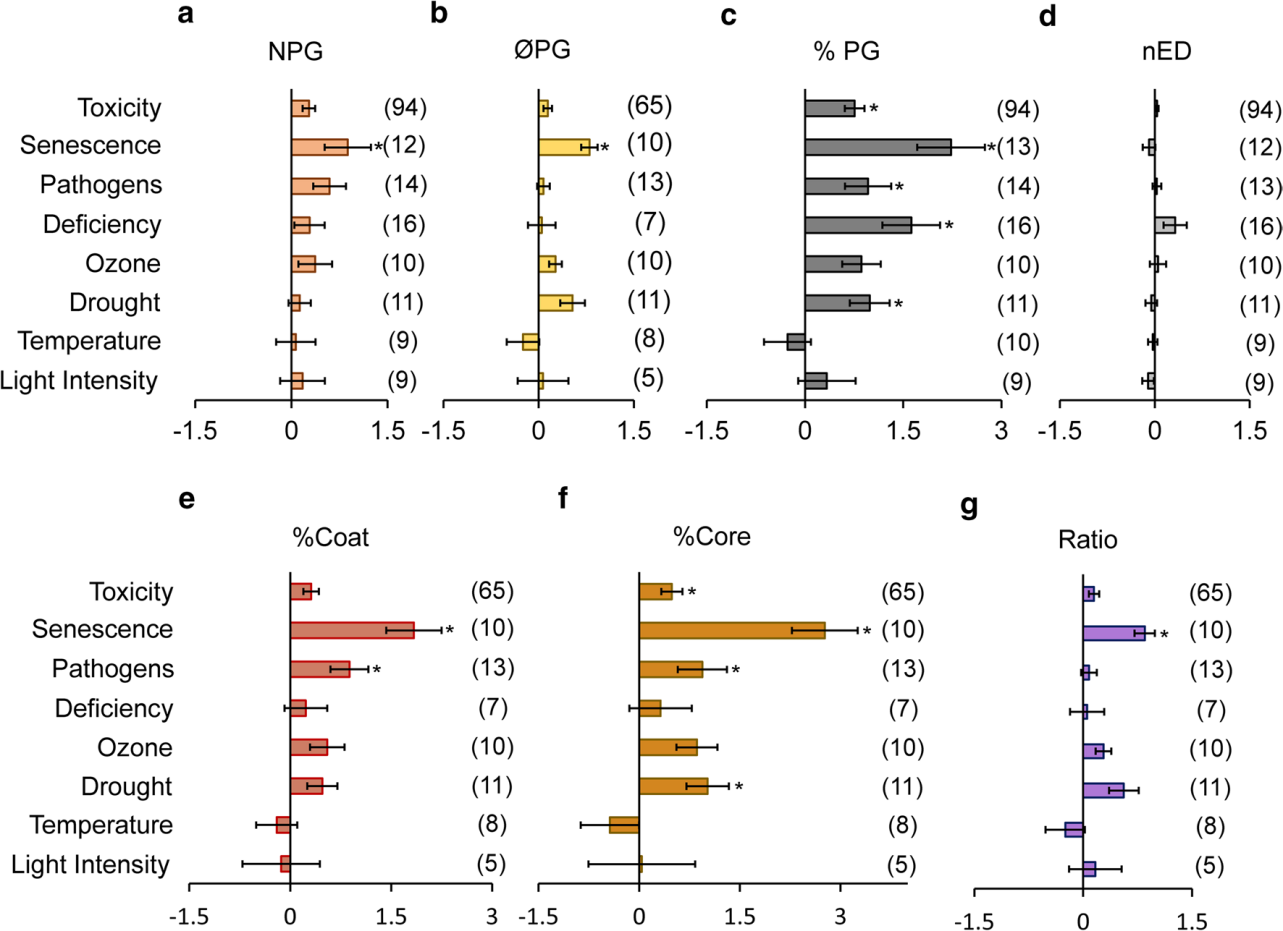
Effect sizes of the main PG parameters under specific stress. **a** Number of PGs per chloroplast cross-section (NPG). **b** Diameter of PG (ØPG). **c** Percentage of the chloroplast cross-section area occupied by PG (%PG). **d** Normalised electrodensity (nED). **e** Percentage of the chloroplast area occupied by total coat area (%Coat). **f** Percentage of the chloroplast area occupied by total core area (%Core). **g** Ratio between %Core and %Coat. Data were calculated as: ln(X_*Stress*_/X_*Control*_). Each value was considered non-stress or stress based on the criteria of each article’s authors. Notice that the difference on the number of cases among parameters is due to the availability of a size scale in the pictures and to the quality of the image. Bars and whiskers represent the mean ± SE (*n* specified within brackets for each stress). Asterisks indicate statistically significant differences between non-stress and stress values (*P* < 0.05)

For those stressors reported in more than ten studies, we evaluated the effects in all parameters in more detail by separating the results per stress type (Fig. 6). Except for temperature and light intensity, all the other stress types induced a positive response of the parameters. Among the rest, senescence presented the strongest effect in most of the parameters, followed by drought, pathogens and ozone. On the other hand, the %PG was the only parameter that experienced a statistically significant rise on almost all stressors (Fig. 6c). The increase in the %Coat (Fig. 6e) was statistically significant for senescence and pathogens, whereas in the case of %Core (Fig. 6f), it was for toxicity, senescence, pathogens and drought. In contrast, nED was notably unaffected by any stressor (Fig. 6d), with the exception of nutrient deficiency that caused an increase in osmiophilicity. Moreover, the extent of response in the seven parameters differed among stressors. For example, the responsivity of NPG to toxicity and pathogens was higher than that of ØPG for the same stressors (Fig. 6a, b), whereas the opposite pattern was observed in response to drought stress.

We also evaluated whether or not plant taxonomic group determined the response to stress. For this purpose, we compared the response of the seven parameters to toxicity on monocots vs. dicots (Supplementary Fig. S4). This stress was selected as example because it included the largest dataset among all factors considered in this study. The results showed no significant differences (either in direction or magnitude) between mono- and dicots.

**Fig. 7:**
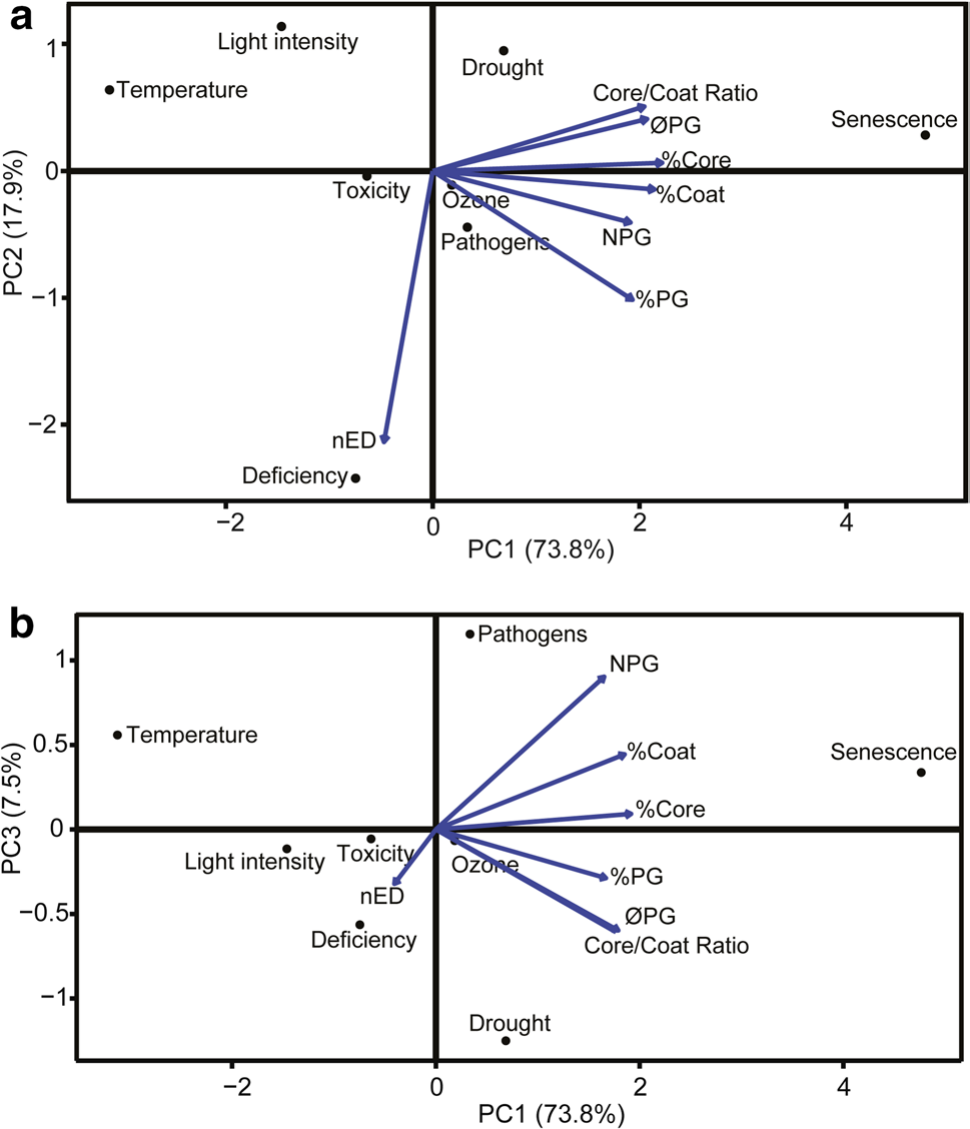
Biplot of the principal component analysis (PCA) of PGs attributes and stress types. **a** PCA biplot displaying the effect of PC1 and PC2. **b** PCA biplot displaying the effect of PC1 and PC3. The percentages refer to the amount of data variability explained by each component. Blue arrows point in the direction of PGs attributes maximum variation. nED, normalised electrodensity; NPG, number of PGs per chloroplast cross-section; Ratio, %Core/%Coat ratio; ØPG, diameter of individual PG; %Coat, percentage of chloroplast cross-section area occupied by the total coat area; %Core, percentage of chloroplast cross-section area occupied by the total core area; %PG, percentage of chloroplast cross-section area occupied by the total PG area

Finally, to better visualise this responsiveness, the effect of each stress on PGs was displayed by a PCA based on the effect size of each attribute (Fig. 7). The analysis showed that PC1 explained most of the data variability (73.8%), while PC2 explained 17.9% and PC3 7.8%. The first component separated senescence from the rest of stress types, being mostly positively related to PGs morphological changes (except for nED), while temperature also differed from the rest, correlating negatively with PC1. Nutrient-deficiency was separated from the other stresses by the second axis (PC2), which is directly related to nED (Fig. 7a). Finally, in the PC3 axis, stressors were separated based on NPG or ØPG, being “pathogens” the stressor more closely linked to NPG, and drought the one more related to ØPG (Fig. 7b).

## Discussion

During the 50s, and in concomitance with the first chloroplast TEM image analyses, Hodge et al. (1955) described what they denominated “*small* *dense* *spherical* *bodies*”. These structures are nowadays known as plastoglobules. For more than 50 years, their physiological role remained hidden, and it was only during the last decade, when their functions were highlighted after the characterisation of their proteome (Vidi et al. 2006; Ytterberg et al. 2006; Lundquist et al. 2012). By the novel approach used in the present study, namely the quantitative re-evaluation of published TEM images, we present in numbers the morphological plasticity of PGs in functional chloroplasts. Nevertheless, we are aware that even though the image selection and analyses were performed in a systematic way, bias can be originated from an inaccuracy in the analysis of the images, the image selection criteria of the authors of each article (selecting eye-catching images rather than representative ones) or the lack of studies regarding certain plant divisions or stress types. Furthermore, the quantitative values obtained correspond to the two-dimensional (2D) projection of an originally three-dimensional structure created by randomly generated cuts. Despite it, the present study (i) uncovers some of the common attributes of these subcompartments under non-stress conditions, (ii) determines some of their main morphological changes in response to stress (including in which direction) and (iii) identifies some underexplored aspects of PGs that should be addressed still further, i.e. their attributes within Cryptogamic taxa, their changes in electron density, or the potential relationship between their size and function.

### PGs in non-stressed chloroplasts: characterisation

The picture of the most frequent type of PG from a non-stressed chloroplast depicts a spherical structure, with a diameter of around 100–200 nm, representing less than 3% of the chloroplast area (Fig. 3). In agreement with the values reported in several reviews (Lichtenthaler 2007; Nacir and Bréhélin 2013; van Wijk and Kessler 2017), the PGs diameter was in the range 50–200 nm for 65% of the cases. However, our analysis provides examples of larger diameters, probably linked to species-specific traits. This is the case of species such as *Fagus* with ØPG up to 900–1500 nm (Steinmüller and Tevini 1985; Mikkelsen and HeideJorgensen 1996) or *Chromolaena* *odorata*, only reported once, with ØPG up to 1400 nm in non-stress conditions (Raman et al. 2006). Furthermore, nED was uniformly high in PGs under non-stress conditions (Fig. 3d). Considering that osmiophilicity is directly related with the composition of PGs due to the interactions of osmium tetroxide with the double bonds of plastoquinone, carotenoids and triacylglycerides, as well as the reducing power of tocopherols and plastoquinone (Singh et al. 2010), electrodensity of PGs is very likely related with an specific chemical composition involving these molecules.

The relationships of the attributes indicated that two different “populations” of PGs could be present in the chloroplasts (Fig. 4, Supplementary Fig. S3): one composed of a high number of small/medium-sized PGs (< 300 nm), and a second one formed by a reduced number of large-sized PGs (> 300 nm). A third intermediate “population” could also be considered, characterised by several medium-sized PGs. The increase of either attribute (number or diameter) is directly related to the increase of the coating lipid monolayer or the central core, respectively. The composition of each part is different, with the proteins and amphipathic lipids only present in the coat and with neutral lipids in the core. Such differences in the lipid/protein ratio may result in different densities of PGs (Bailey and Whyborn 1963; Kessler et al. 1999; Vidi et al. 2006), as suggested by Kessler et al. (1999). Therefore, based on the fact that a higher proportion of coat (result of a higher number of PGs) may favour the accumulation of proteins, we hypothesise that these PGs of different densities could correspond to the “populations” explained above. However, none of the mentioned studies analysed separately which PGs morphology corresponded to each density.

While there is a huge amount of references supporting the positive response of PGs size and number to environmental stresses, their presence in chloroplasts from non-stressed plants has received much less attention. Our study supports that their presence is likely constitutive, with only a few cases (4.5%) where PGs were not observed (Fig. 3). In the instances where no PG was found, it cannot be excluded that other cross-sections of the chloroplast would present at least one PG. Moreover, the minimum diameter reported in our analysis was of 20 nm. In accordance with a core/coat ratio always superior to one, this may indicate that a minimal diameter is required to form a stable spherical structure from the curvatures of the thylakoid grana (Austin II et al. 2006). Likewise, there was not a linear relationship between the NPG, ØPG or %PG and the chloroplast cross-section area (Fig. 4, Supplementary Fig. S3). This indicates the absence of requirement for a minimum PG proportion for the correct functioning of a chloroplast. Alternatively, it could be hypothesised that the quantity of PGs is likely related to the thylakoid amount, rather than to the whole chloroplast cross-section area, because of their structural connection that enables a dynamic bidirectional interaction (Austin II et al. 2006; Eugeni Piller et al. 2012). The resolution of the analysed images hampered the determination of the thylakoid membrane area, and therefore, this hypothesis could not be proved in this study.

### PGs in response to stress: implications in morphology and composition

**Fig. 8:**
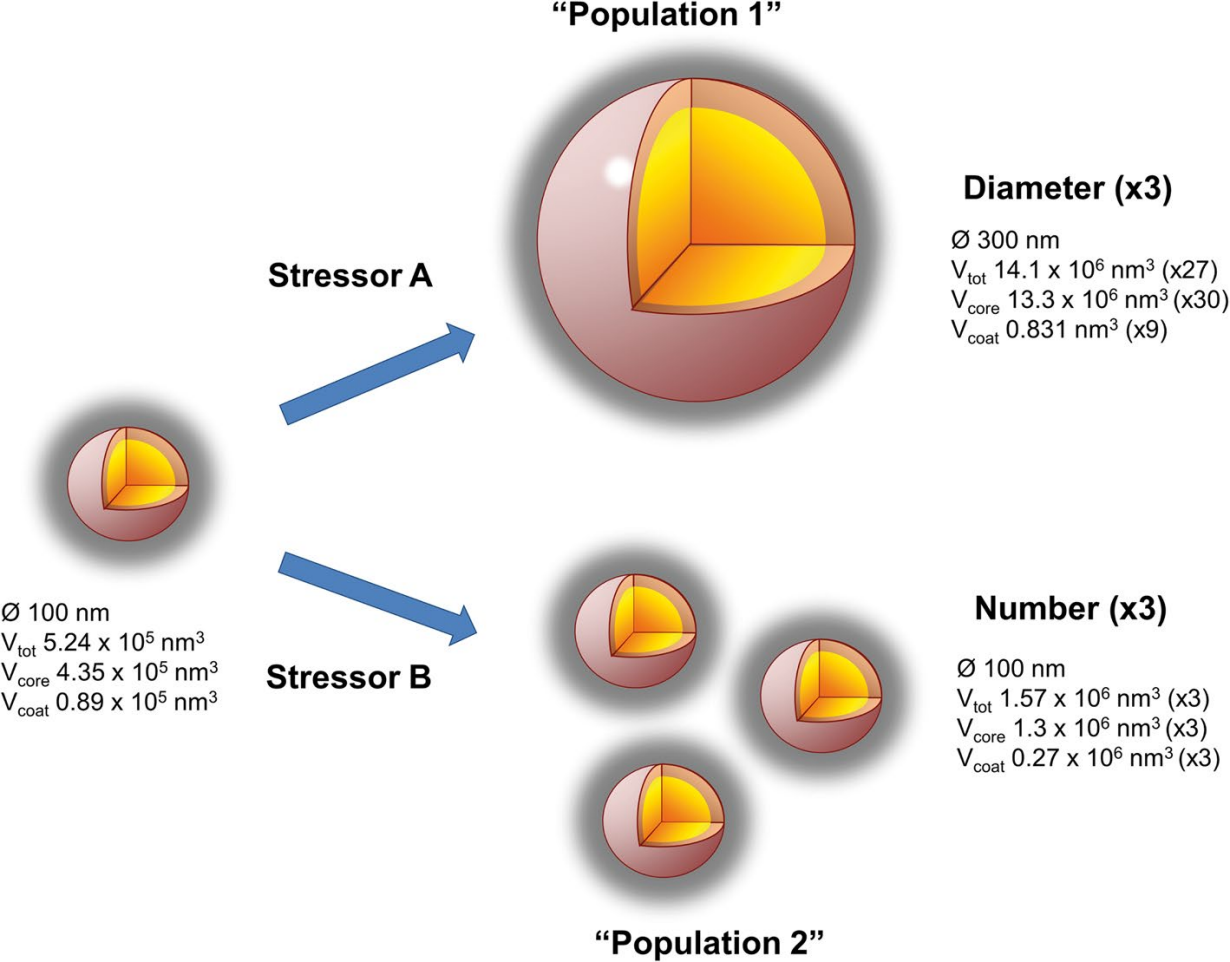
Model of plastoglobules stress-dependent morphological changes. When the plant is subjected to different stressors, the triggered response increases the diameter (“Population 1”) or the number (“Population 2”) of PGs. This causes a different modification of the core/coat ratio, and therefore, an alteration on the proportions of their composition. The number in parenthesis indicates the increment of the volume

Chloroplasts have an enormous capacity to cope with and adapt to changing environmental conditions. These responses involve both biochemical and ultrastructural modifications (Kirchhoff 2014). Among the latter, changes in PGs are the most noticeable. In our analysis, these were reflected by %PG, the most responsive parameter (Figs. 5, 6). The increase in %PG can be caused by a higher number and/or diameter of PGs, thereby altering the core/coat ratio. Consequently, these alterations in PGs morphology might be directly reflecting changes in their composition, as suggested by Lundquist et al. (2013). Thus, an increase in the diameter would denote an increase of neutral lipids likely resulting from thylakoid remodelling, while a rise on the number of PGs would imply more space for accumulation of proteins and prenyl lipids (Fig. 8). For example, the expansion of the diameter from 50 to 100 nm would cause a higher increase in %Core (4.6-fold) than the %Coat (2.1-fold), raising up the core/coat ratio 2.2-fold. Whereas the increase in the number of PGs from 10 to 20 would maintain the ratio constant at 3.4, implying a similar accumulation of the components of both parts.

Therefore, we hypothesise that the specific alterations of their morphology would also be cause and consequence of a change in the abundance of the different components. This relationship between morphology and composition is supported by previous studies with mutants overexpressing certain genes involved in PG metabolism and organisation. For example, overexpression of VTE1 in Arabidopsis generated the accumulation of PC-8, along with an increase in the number of small PGs (Zbierzak et al. 2010). In tobacco, the overexpression of FBN1 also derived in an increase of the number of PGs (Rey et al. 2000). By contrast, drought stress caused the opposite effect, leading to an increase in their diameter both in the WT and in the overexpressing-FBN1 mutant (Rey et al. 2000). Changes in metabolites were not evaluated in this work, but it can be presumed that the increase in the core would trigger the accumulation of neutral lipids, as has been observed in senescence (Kaup et al. 2002). This process probably relates to the remodelling/dismantling of the components of thylakoids in response to drought (Da Silva et al. 1974). In addition, Arabidopsis plants exposed to high light stress shifted differently in PGs morphology for WT and *abc1k1/abc1k3* mutant (Lundquist et al. 2013). In the WT the PGs became larger, whereas in the mutant, the number of PGs increased. Subsequent metabolite analysis for the exact same light stress in WT Arabidopsis reported a remobilisation of carotenoids to PGs (Espinoza-Corral et al. 2021), which could be related to the increase in the diameter reported in Lundquist et al. (2013).

In addition to general stress response, the specific response of PGs attributes to each stressor was also analysed (Figs. 6, 7). The results revealed that the response level of PGs from senescing chloroplasts, the so-called gerontoplasts (Keskitalo et al. 2005), differed considerably from the PGs of plants affected by the other types of stresses, probably due to the organised pattern of modifications that leaves undergo during senescence. Among the rest, we observed that the level and type of change in the attributes varied depending on the stressor. Three main response types, that directly related attribute changes with specific stressors, were evidenced by the PCA (Fig. 7): the increase of NPG with pathogens, the increase of ØPG with drought and the alterations in nED with nutrient deficiency. Therefore, the increase on number or diameter (and thus the change in the core/coat ratio) may be tightly related to the specific stress that the plant undergoes (Fig. 8). This also suggests that stress-specific responses of PGs morphology lead to a particular alteration on their composition. Thus, changes in particular components have been observed in response to a specific stress as is the case of high light stress, that induces little but specific alterations in proteins and prenyl lipids (Espinoza-Corral et al. 2021). In addition, changes in specific FBNs can be observed upon different stressors (Singh and McNellis 2011), and relate to specific metabolic pathways, depending as well on plant tissue type (Michel et al. 2021).

In the particular case of temperature, cold and hot treatments were considered together, but no specific trend was observed in any of the parameters if considered apart. Indeed high variability in the responses was obtained both within cold and within hot treatments (data not shown). In addition, the amount of values under the stress “temperature” was very low (*n* = 10). Since membranes change their composition to maintain the optimal fluidity for the correct function of the proteins within it (Martinière et al. 2011) and based on the dynamic connection of PGs and the bilayer (Austin II et al. 2006), we presume that the change in the composition of the bilayer could also affect the PGs. This is not supported by the obtained results, suggesting that the regulation of fluidity of the membrane may not be directly connected to the PGs. Nevertheless, more data are needed before a solid conclusion can be drawn.

Electrodensity is one of the least considered attributes in PGs in the literature. We have found only some examples where the differences in electrodensity were reported in the text (Steinmüller and Tevini 1985; Tevini and Steinmüller 1985; Lundquist et al. 2013), but none in quantitative values. This may be related to the fact that it is the attribute with the lowest responsiveness to stress (Figs. 5, 6). However, while the other attributes did not change remarkably, the variance on electrodensity was notable in response to nutrient deficiencies. This is probably induced by the alterations of metabolism caused by the deficiency of essential elements, and therefore changes in metabolites, as has been reported for the case of nitrogen (Gaude et al. 2007), phosphate (Pfaff et al. 2020) or magnesium (Yang et al. 2019). However, the results obtained from this parameter have to be considered with caution because the obtained data could be affected by the technical processing of the samples during the acquisition of the image, as it highly depends on the procedure of fixation, staining and contrast parameters employed.

Finally, PGs tend to generate from the thylakoid curvatures or from other PGs, forming grape-like clusters (Austin II et al. 2006; Zbierzak et al. 2010). The presence of PGs connected to each other was considerably high. In 62% of the analysed chloroplasts at least one pair of PGs were attached to each other (data not shown). The number of clusters did not discern significantly between non-stress and stress conditions (a mean value of 1.5 and 1.55 clusters respectively). By contrast, grape-like clusters with more than 10 PGs were mainly found in stress conditions (approximately 460 clusters in non-stress over 1070 clusters in stress conditions). The generation of these clusters can be interesting mainly in the cases where the number of PGs increases strongly, because interconnected network of PGs could be beneficial to cope with the stress. It was also remarkable that some of these clusters were organised in a line fitted between thylakoid membranes, showing a specific organisation within the chloroplast.

Overall, the novel approach of the present quantitative review confirms that PGs are ubiquitous subcompartments in chloroplasts under non-stress and stress conditions and that PGs might be used as an indicator of stress response, as has been proposed by other authors (Polesi et al. 2019; Khan et al. 2021). The study of their composition, mainly the characterisation of its proteome, has been a major breakthrough in the advancement of our understanding on how these subcompartments interact with the whole chloroplast. As their number, size and osmiophilicity are somehow related to their composition and to the development of specific responses to stressors (Fig. 8), a more in depth study of their morphology could provide specific information on the stress type and strength. It could also contribute to set a baseline towards understanding understudied aspects such as the synthesis and disappearance of PGs over time (during daytime or under stress), or possible functional specialisation between PGs in the same chloroplast. Thus, advances regarding PG morphology will eventually help to fulfil our current understanding on how chloroplast perform and acclimate to environmental conditions.

#### *Author contribution statement*

JIG-P planned and designed the research. JIG-P and MIA performed the data search. BF-M, JIG-P and MIA performed the image analysis measurements. MIA analysed the data under the supervision of BF-M and JIG-P. All the authors wrote the manuscript and contributed to subsequent revisions and discussion.

## Supplementary Information

Below is the link to the electronic supplementary material.

Supplementary file1 (XLSX 583 KB) Supplementary data are available at *Planta* online

Supplementary file2 (PDF 3333 KB)

## Data Availability

All the data generated or analysed during this study are included in this published article and its supplementary information files.

## Abbreviations

FBN: Fibrillin
nED: Normalised electrodensity
NPG: Number of PGs per chloroplast cross-section
PG: Plastoglobule
PCA: Principal component analysis
TEM: Transmission electron microscopy
ØPG: Diameter of individual PG
%Coat: Percentage of chloroplast cross-section area occupied by the total coat area
%Core: Percentage of chloroplast cross-section area occupied by the total core area
%PG: Percentage of chloroplast cross-section area occupied by the total PG area
