# Supplementary material for: More than just lipid balls: quantitative analysis of plastoglobule attributes and their stress-related responses
Source: Planta. 2022 Feb 10;255(3):62. doi: 10.1007/s00425-022-03848-9 (PMC8828631; doi:10.1007/s00425-022-03848-9)
Supplement: Supplementary file 2 — Supplementary file2 (PDF 3333 KB) [file 425_2022_3848_MOESM2_ESM.pdf]

Supplementary information

More than just lipid balls: quantitative analysis of plastoglobule attributes and their stress-related responses

Miren I. Arzac[1*], Beatriz Fernández-Marín[1,2], José I. García-Plazaola[1]

[1]Dept. Plant Biology and Ecology, University of the Basque Country (UPV/EHU), Barrio Sarriena s/n, 48940 Leioa, Spain

[2]Dept. Botany, Ecology and Plant Physiology, University of La Laguna (ULL), Tenerife 38200, Spain

* Corresponding author: mirenirati.arzac@ehu.eus

Journal: Planta

Fig. S1 TEM images showing some of the PGs traits analysed in the present study. **a** Small sized (ø<50 nm) PGs in phyllids of the moss *Syntrichia ruralis*. **b** Middle sized (100<ø<200 nm) PGs in leaves of the fern *Hymenoglossum cruentum*. **c** Large-sized (ø>200 nm) PGs in leaves of winter acclimated *Buxus sempervirens*. **d** PGs showing differential osmiophilicity in phyllids of the moss *Syntrichia ruralis* after rapid mild desiccation and subsequent rehydration. **e** Clusters of PGs (pearl necklace-like) in phyllids of the moss *Syntrichia ruralis* after rapid mild desiccation and subsequent rehydration. **f** Clusters of PGs (grape-like) in phyllids of the moss *Syntrichia ruralis* after rapid desiccation and subsequent rehydration. Bar size = 0.5 µm (**a** and **d**), 0.2 µm (**b**), 1 µm (**c**, **e**, **f**).

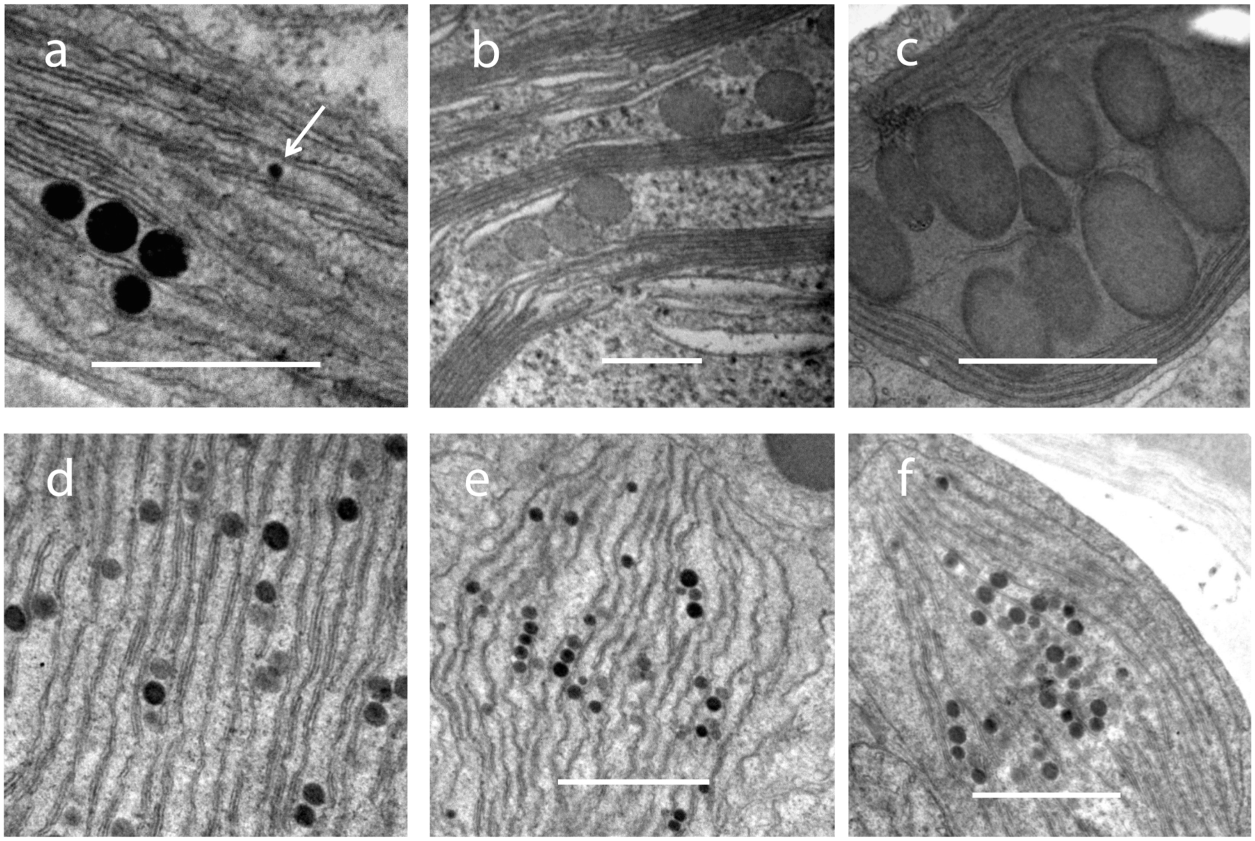

Fig. S2 Relation between numerical data provided by the authors of the article and data obtained by us through image analysis from figures of the same article. **a** Chloroplast cross section area (CA). **b** Total area of PG per chloroplast cross section. **c** Percentage of chloroplast cross section area occupied by the total PG area (%PG). **d** Number of PG per chloroplast cross section (NPG). **e** Diameter of PGs (ØPG). The continued line represents the linear trend line of the data and the dashed line the 1:1 relationship. Statistically significant differences are indicated by the asterisks (*P*<0.05).

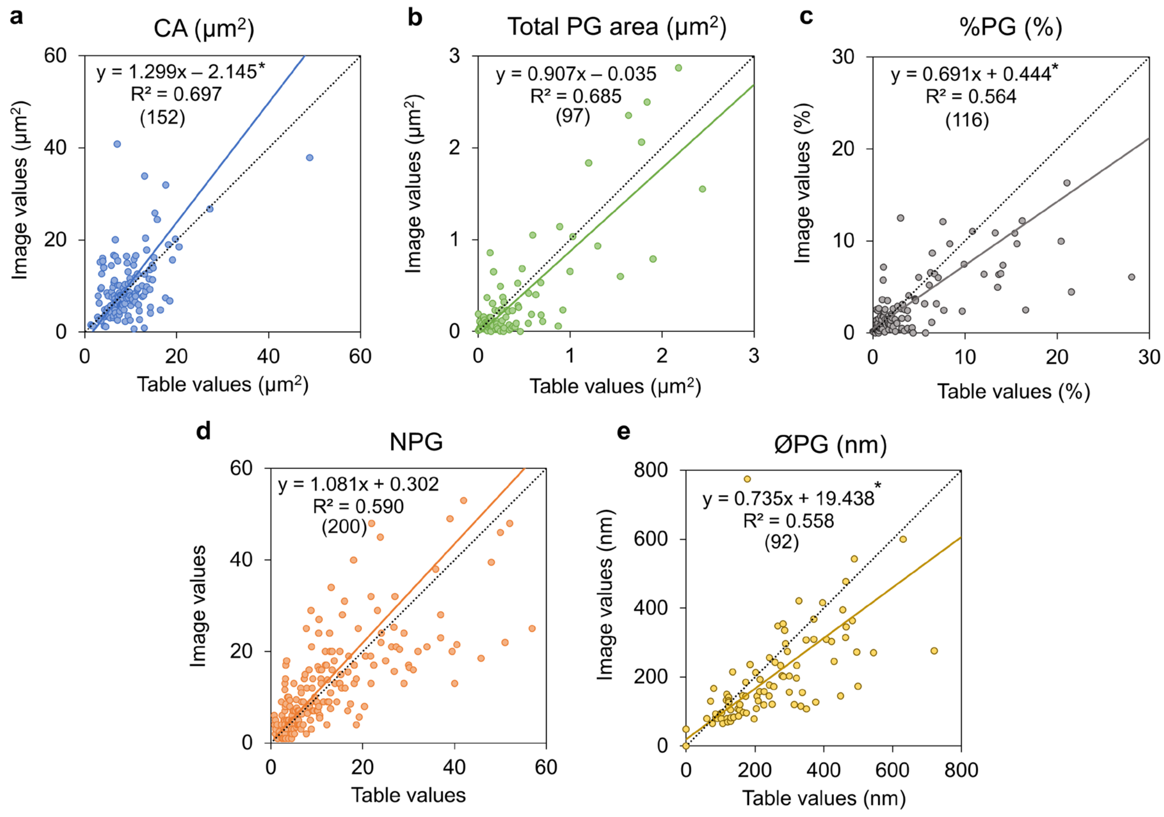

Fig. S3 Correlations between the parameters analysed in the study not shown in Fig. 3. **a** Percentage of chloroplast cross section area occupied by the total PG area (%PG) compared to chloroplast cross section area (CA). **b** Normalised electrodensity (nED) compared to CA. **c** Percentage of chloroplast cross section area occupied by the total core area (%Core) compared to CA. **d** Percentage of chloroplast cross section area occupied by the total coat area (%Coat) compared to CA. **e** Diameter of PG (ØPG) compared to nED. **f** ØPG compared to %PG. **g** ØPG compared to %Core. **h** %PG compared to nED. **i** Number of PGs per chloroplast cross section (NPG) compared to %PG. **j** %PGs compared to %Core. **k** nED compared to %Core. **l** NPG compared to %Coat. **m** % PGs compared to %Coat. **n** nED compared to %Coat. **o** %Coat compared to %Core. The circles represent the values of stress conditions and the squares the values of non-stress conditions.

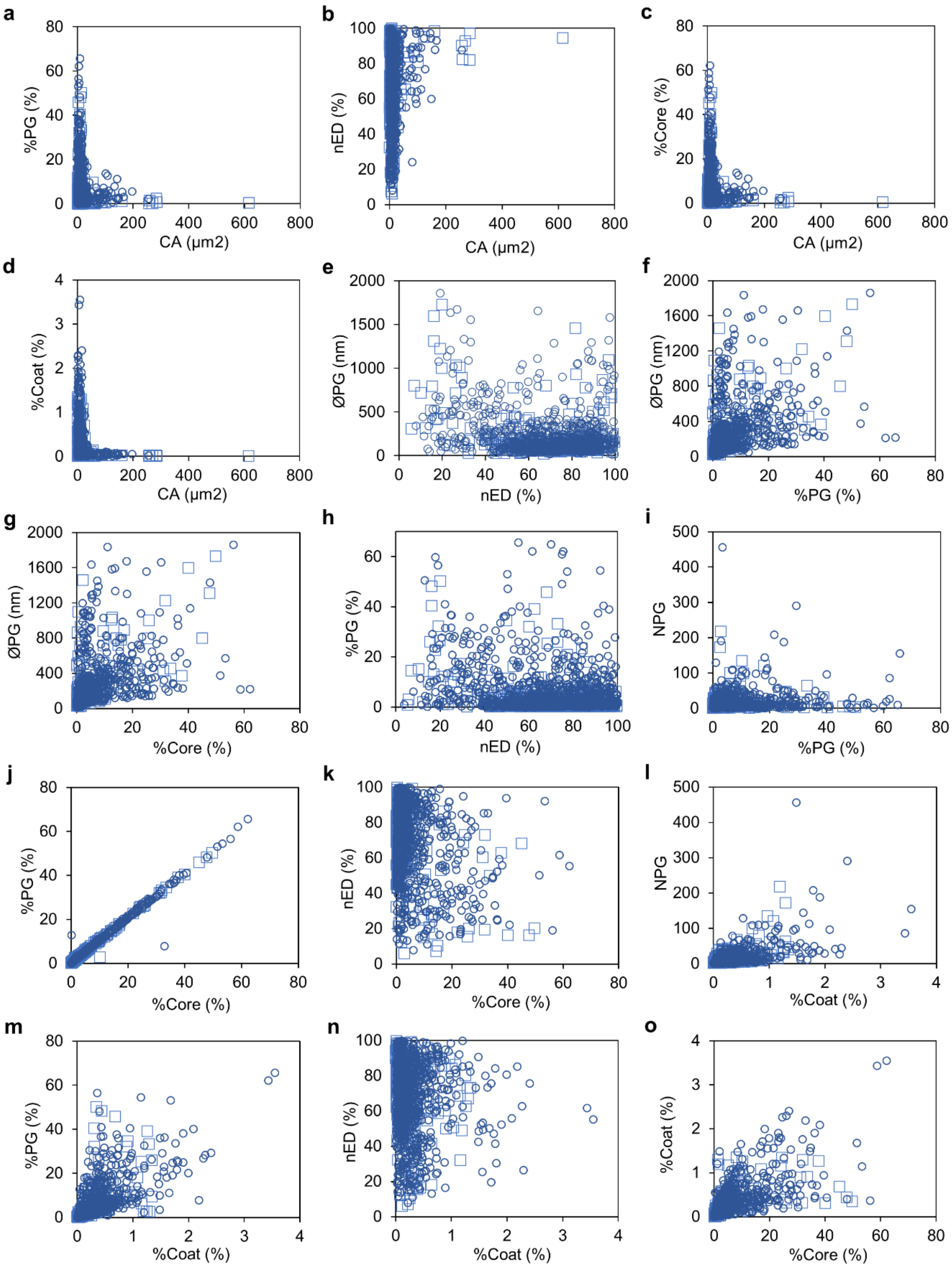
a
%PG (%)
CA (μm2)
b
nED (%)
CA (μm2)
c
%Core (%)
CA (μm2)
d
%Coat (%)
CA (μm2)
e
ØPG (nm)
nED (%)
f
ØPG (nm)
%PG (%)
g
ØPG (nm)
%Core (%)
h
%PG (%)
nED (%)
i
NPG
%PG (%)
j
%PG (%)
%Core (%)
k
nED (%)
%Core (%)
l
NPG
%Coat (%)
m
%PG (%)
%Coat (%)
n
nED (%)
%Coat (%)
o
%Coat (%)
%Core (%)

Fig. S4 Comparison of the response to toxicity between dicot and monocot species. nED, normalised electrodensity; NPG, number of PGs per chloroplast cross section; Ratio, %Core/%Coat ratio; ØPG, diameter of individual PG; %Coat, percentage of chloroplast cross section area occupied by the total coat area; %Core, percentage of chloroplast cross section area occupied by the total core area; %PG, percentage of chloroplast cross section area occupied by the total PG area.

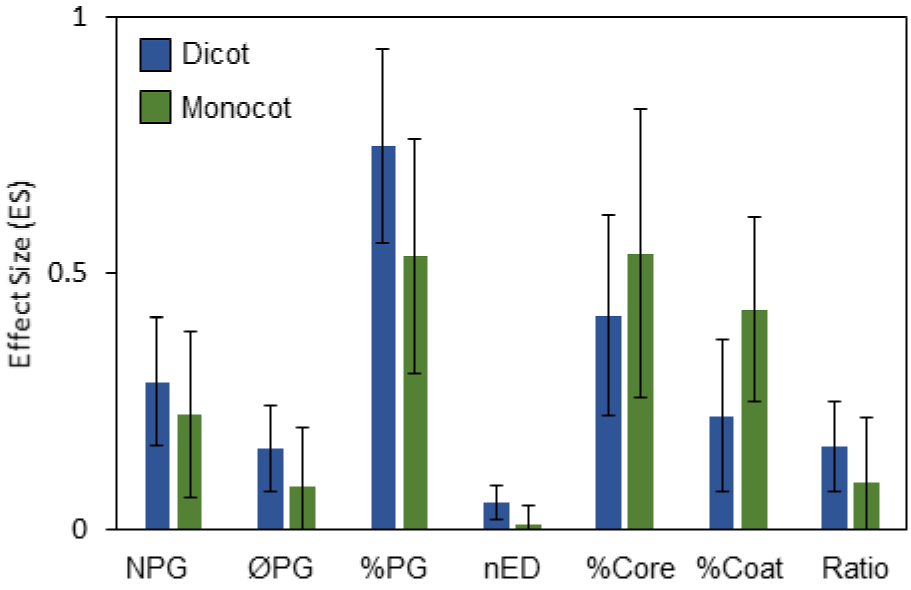

Supplementary Table S1. Description of the different stress types present in this analysis.

Stress type	Description	Examples
**Toxicity**	Any damaging external substance applied to the plants.	*heavy metals*: lead, copper, silver, cadmium, chromium, titanium, boron, yttrium, nickel…<br>*NaCl or sea water* (50mM, 100mM, 200mM…)<br>*herbicides*: glyphosate, paraquat, paclobutrazol, atrazine, DTBP, fusillade, DCMU, isoxaflutole, diurion, difenzoquat…<br>*other substances*: caffeine, phosphite, phosphate, industrial ash, mine soil, contaminated water, SO_2, NO_X, NaF, NH_4NO_3...
**Senescence**	The last stage of the development, when a coordinated degenerative process takes place.	Differences among a time period (days to weeks)
**Pathogens**	Any damaging pathogen or toxin released by the pathogens	*fungi*: *Botrytis cinerea, Capnodium, Acremonium zonatum, Cercospora beticola…*<br>*virus*: plum pox virus (PPV)…<br>*insects*: *Nothotrioza*, gall…
**Deficiency**	Deficiency of an essential element for the plant.	*nitrogen shortage:* 125mmolN/m^2 , 30% or 0% of the original dose, 40ppm or 120ppm…<br>*magnesium shortage:* 30% or 0% of original dose…<br>*phosphorous shortage:* 30% or 0% of original dose<br>*boron shortage*<br>*calcium shortage*<br>*potassium shortage*<br>…
**Ozone**	Application of elevated ozone levels on plants.	50ppb, 100ppb, 150ppb…<br>50-100 µl l^{-1} h^{-1}<br>...

**Drought**	Application of a shortage of water availability to plants.	*control*: well watered or -1.6MPa<br>*drought:* PEG application, 7-10% of control watering, -4MPa, 50 or 70%RWC, total withdraw of water for an specific period of time (hours, days, weeks…)…
**Temperature**	Exposition to sub- or supra-optimal temperatures	*low temperature:* 4ºC for 2 hours, 5ºC, 10ºC, gradual decrease from 24/15ºC to 9/4ºC, gradual decrease from 23/15ºC to 8/1ºC, 2ºC during 10 days…<br>*high temperature:* 40ºC for 40 min…
**Light Intensity**	Overall changes in light intensity, either high or low irradiance.	30%, 50% or 100% of max PFD<br>40, 50, 100, 200, 400 µmol m^{-2} s^{-1}<br>30, 60 or 90% of shadow<br>…
**Plant development**	Studies where the plant was analysed during different developmental stages, in the absence of any additional stress factor.	Comparison of plant leaf expansion stages<br>Comparison of young or old leaves<br>Differences among ages (1 year vs. 5-6 years)<br>…
**Seasonal variation**	Observation of seasonal-related changes in morphological traits	Spring, summer, autumn, winter
**Desiccation/rehydration**	Application of a shortage of water availability to plants that can tolerate a loss up to 95% of their water content and recover afterwards (resurrection plants).	*dehydration:*6 days, 8 days, 9 days, 13 days…<br>*rehydration*: 1 hour, 2 hours, 4 hours, 8 hours, 12 hours and 24 hours…
**Elevated CO_2**	Application of elevated levels of CO_2 (e.g. above ambient concentration)	*control*: around 350- 400ppm<br>*stress*: 600-750ppm
**Others**	Other types of stressors not included in the previous categories.	Age, high altitude, chlorosis, growth conditions, genotype, acid rain…
